# Radiological Findings in Patients with COVID-19

**DOI:** 10.7759/cureus.7651

**Published:** 2020-04-12

**Authors:** Sahar Fatima, Iqbal Ratnani, Maha Husain, Salim Surani

**Affiliations:** 1 Critical Care, Houston Methodist Hospital, Houston, USA; 2 Critical Care Medicine, Debakey Heart and Vascular Center, Houston, USA; 3 Internal Medicine, Dow International Medical College, Karachi, PAK; 4 Internal Medicine, Texas A&M Health Science Center, Bryan, USA; 5 Internal Medicine, Corpus Christi Medical Center, Corpus Christi, USA; 6 Internal Medicine, University of North Texas, Dallas, USA

**Keywords:** coronavirus pandemic, covid-19, radiological findings

## Abstract

After its origin in Wuhan, China, coronavirus related respiratory illness spread across the globe, being declared as a pandemic by WHO on March 13, 2020. Because it is acquired via respiratory droplets, community spread is responsible for the recent global crisis. The current diagnostic options include real-time polymerase chain reaction (RT-PCR) and a few serology tests, including but not limited to the recently approved five minutes serology tests. The disease presents as a lower respiratory tract illness. Anecdotal experiences have shown that imaging characteristics are crucial to diagnosis as radiological evidence of disease appears prior to clinical manifestations and tends to evolve over time, which can be useful in predicting the stage of the disease. CT scan is more sensitive than a chest X-ray in highlighting these changes.

## Introduction and background

Affecting 1,099,389 people and claiming 58,901 human lives [1], the coronavirus related pandemic is one of the deadliest known epidemics in recent times. These numbers are increasing exponentially and, with no definitive treatment or available vaccine in sight, creating havoc for the health and financial systems of the world. 

The earliest reported cases were in Wuhan, the capital city of Hubei province in China. These cases were treated as pneumonia of an unknown origin. As the disease spread, China alarmed the World Health Organization (WHO) of the presence in Wuhan of several cases of an unusual type of pneumonia. Researchers discovered that the pathogen responsible for the respiratory illness was a novel strain of the family Coronaviradae, which is similar to two previous epidemics, namely Middle Eastern Respiratory Syndrome (MERS) and Severe Acute Respiratory Syndrome (SARS). This new illness was named SARS-CoV-2 by the International Committee on Taxonomy of Viruses (ICTV) on February 11, 2020. The WHO officially labeled the disease caused by SARS-CoV-2 as COVID-19 in the International Classification of Diseases (ICD) [2].

## Review

Coronavirus infection, although initiated by bat-to-human spread, is now mainly acquired through human-to-human spread (i.e., community spread) via contact, respiratory droplets, and also airborne being contemplated [3]. The infection mainly targets the respiratory system. The spike protein, also known as the S protein, binds to the angiotensin-converting enzyme 2 (ACE2) receptor expressed in the alveolar epithelium; this pathophysiology explains the predominance of respiratory symptoms [4].

Diagnosis

The immediate diagnosis is extremely critical for initiating treatment and containing the disease spread. Currently available diagnostic modalities include laboratory testing with RT-PCR and imaging with chest X-ray and CT scan. Laboratory testing mainly relies on PCR done on samples obtained from the upper and lower respiratory tract and was approved by the Center for Disease Control (CDC) on March 3, 2020 [5]. RT-PCR is highly specific (i.e., 95-97%) but has a low sensitivity of 60-70% [6, 7]. There are a number of serology tests available, and on March 27, 2020, the Food and Drug Administration (FDA) approved a five minutes serology test kit [8].

**Figure 1:**
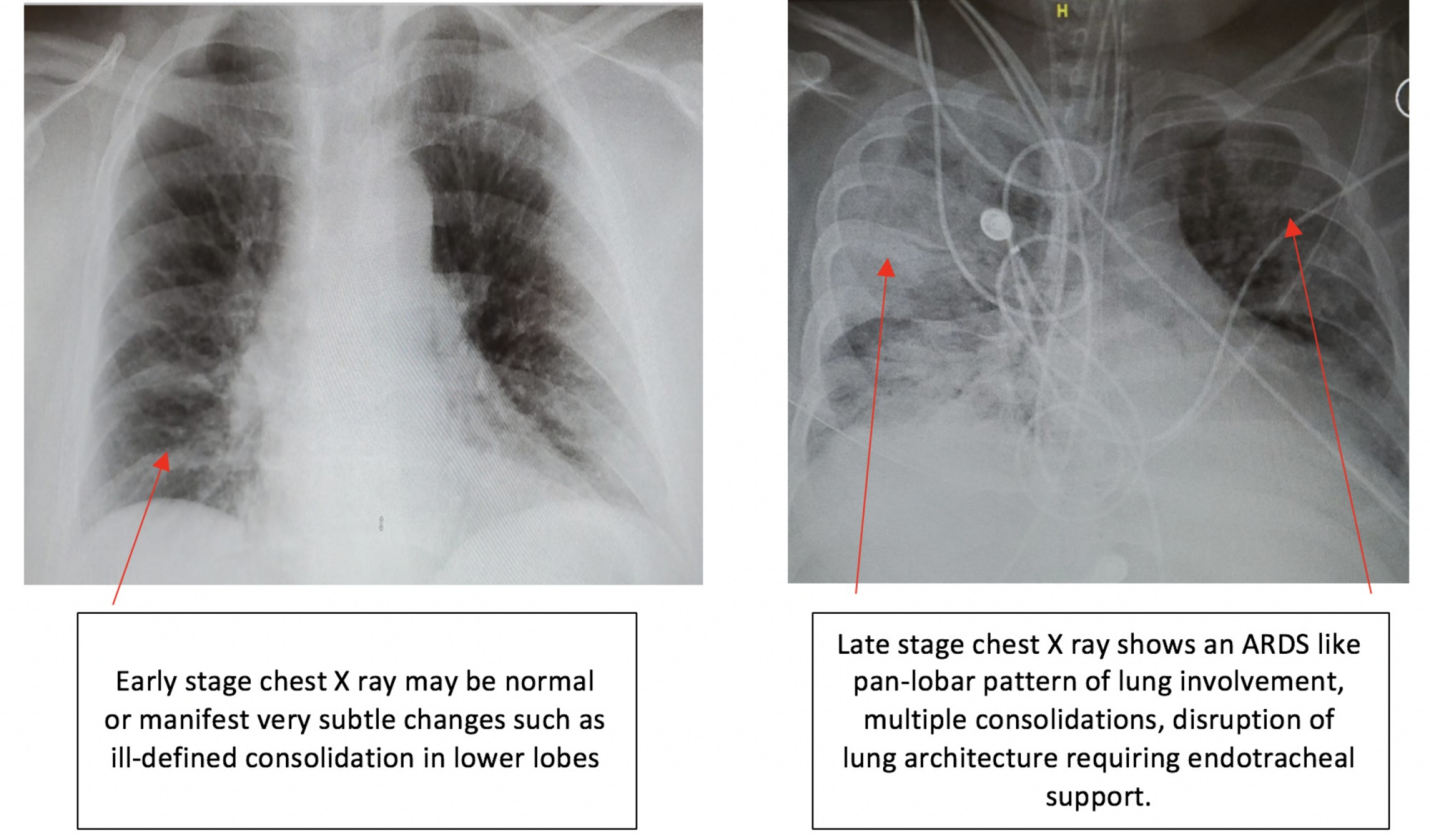
Early and late-stage X-ray findings in patients with COVID-19 ARDS - acute respiratory distress syndrome

The radiological perspective used for disease assessment and follow-up is very helpful. It provides a direct insight into the pathophysiology of the disease process. As the coronavirus related respiratory illness presents clinically as pneumonia, predominant imaging findings are that of an atypical or organizing pneumonia [7, 9]. Although chest X-rays are less sensitive than CT scans, the former may be used as a first-line approach because of their availability and ease of decontamination. Chest X-ray findings may be normal earlier in the clinical course and tend to peak 10-12 days after the onset of clinical symptoms [10]. Figure 1 represents early and late-stage X-ray findings in patients with COVID-19.

**Figure 2:**
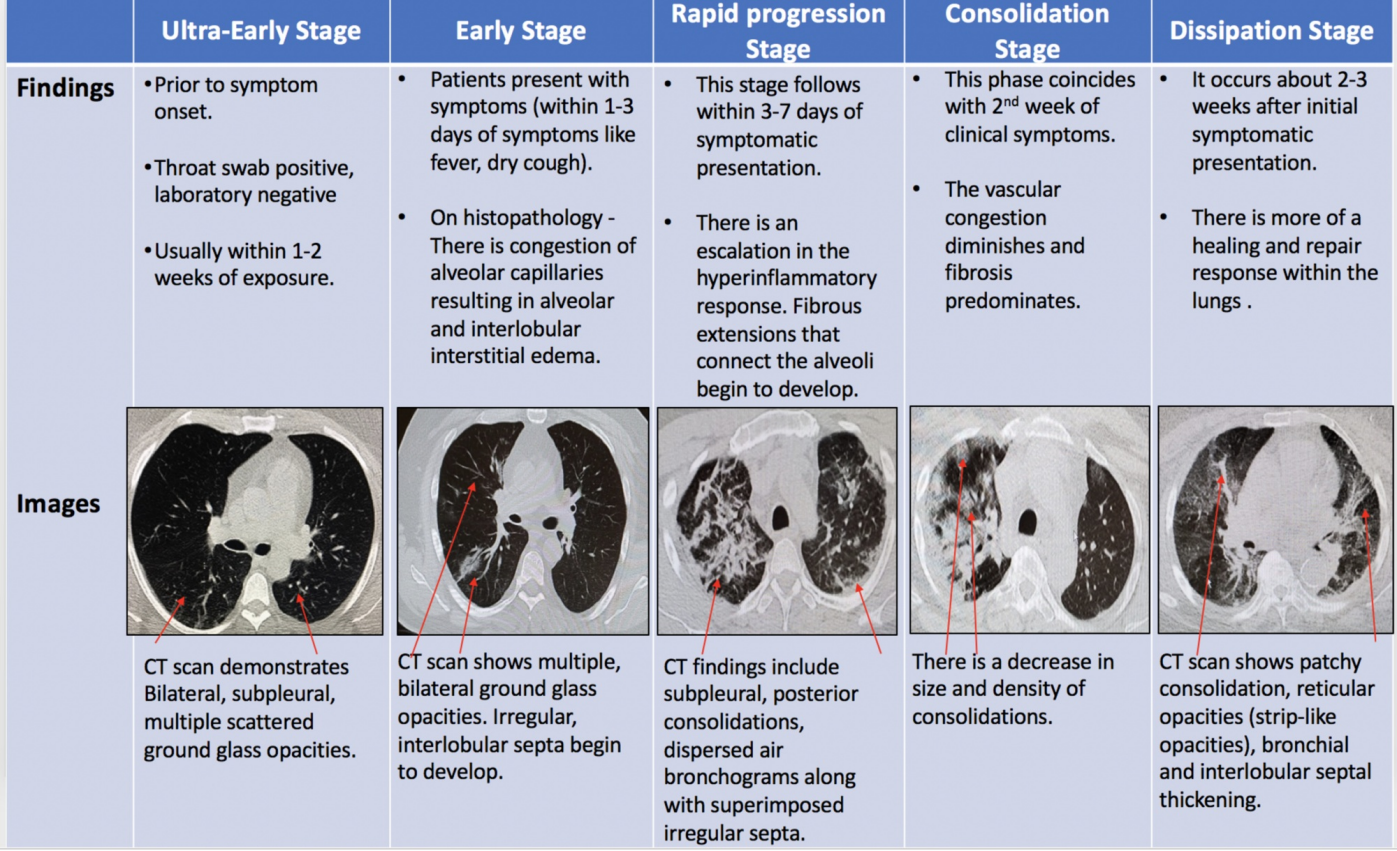
CT scan stages in patients with COVID-19

Chest CT has greater sensitivity as compared to RT-PCR in diagnosing COVID-19 [11]. There has been a lot of debate on using a CT scan as the front-line screening tool for diagnosing COVID-19 [10]. In fact, CT findings begin to appear even before a positive COVID-19 lab result [12]. According to Jin et al., the characteristic CT scan findings evolve in five different phases, as shown in Figure 2 [13].

Lung ultrasound

According to Soldati et al., lung ultrasound (LUS) can detect changes in superficial lung parenchyma with greater accuracy as compared to chest X-rays and can, therefore, play an important role in triage, diagnosis and prognostic stratification of patients in the emergency room (ER) and intensive care unit (ICU) setting [14]. Poggiali and her colleagues recently published a report that suggests that ultrasound findings in patients with COVID-19 pneumonia correlated with CT findings, which signifies that LUS could be useful for early diagnosis of COVID-19 pneumonia in patients presenting to the ER [15].

**Figure 3:**
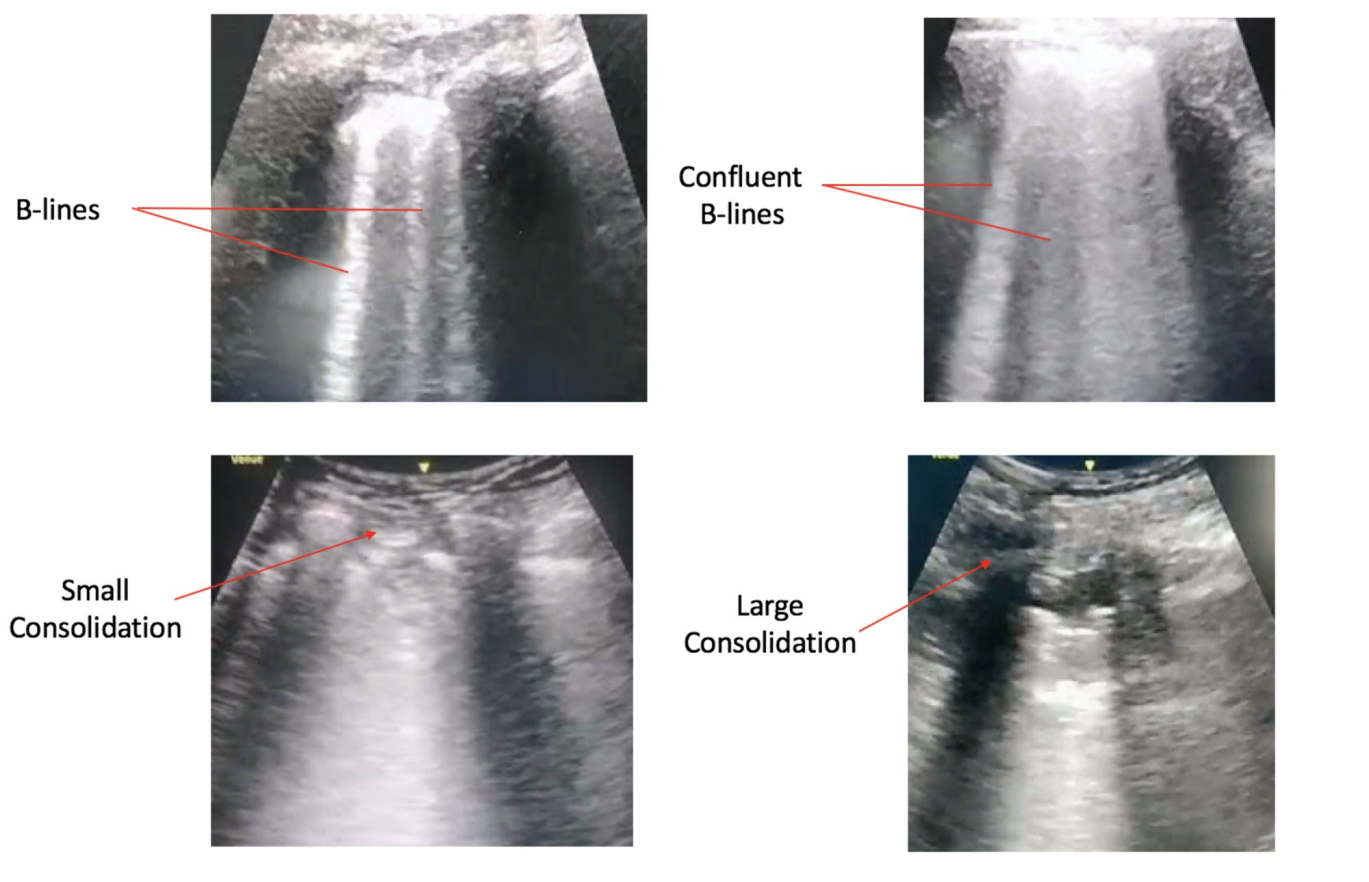
Lung ultrasound findings in patients with COVID-19

## Conclusions

Although most of the patients completely recovered from the disease, they are likely to have some kind of long- term lung damage. Only time will reveal the magnitude of irreversible parenchymal lung injury. Radiological findings are very helpful in predicting the clinical course of the disease and may be used to monitor long-term consequences. There is a lot of research currently being done. With the abundance of new information available, we are observing changing trends in diagnostic and therapeutic approaches to the disease.
